# The *Nothoaspis amazoniensis* Complete Mitogenome: A Comparative and Phylogenetic Analysis

**DOI:** 10.3390/vetsci5020037

**Published:** 2018-03-27

**Authors:** Paulo H. C. Lima, Pedro M. P. Vidigal, Rafael M. Barcelos, Raphael C. Klein, Carlos E. Montandon, Mary H. Fabres-Klein, Jorge A. Dergam, José M. Venzal, Cláudio Mafra

**Affiliations:** 1 Biochemistry and Molecular Biology Department, Federal University of Vicosa, Vicosa 36570-900, State of Minas Gerais, Brazil; paulolima0027@gmail.com (P.H.C.L); maziolirb@gmail.com (R.M.B.); mhfabres@gmail.com (M.H.F.-K.); 2 Federal Institute of Education, Science and Technology of Amazonas, Manaus 69020-120, State of Amazonas, Brazil; 3 Biomolecules Analysis Center, Federal University of Vicosa, Vicosa 36570-900, State of Minas Gerais, Brazil; pedro.vidigal@ufv.br; 4 Multidisciplinary Center of Luís Eduardo Magalhães, Federal University of West of Bahia, Luís Eduardo Magalhães 47850-000, State of Bahia, Brazil; rcontelli@gmail.com; 5 Center for Research in Biological Sciences, Federal University of Ouro Preto, Ouro Preto 35400-000, State of Minas Gerais, Brazil; em.carlos1984@gmail.com; 6 Animal Biology Department, Federal University of Vicosa, Manaus 69020-120, State of Minas Gerais, Brazil; dergam@ufv.br; 7 Department of Veterinary Parasitology, University of the Republic, Montevideo11200, Uruguay; jvenzal@unorte.edu.uy

**Keywords:** argasids, *Nothoaspis amazoniensis*, ticks, phylogenomic, taxonomic arrangement, mitogenome

## Abstract

The molecular biology era, together with morphology, molecular phylogenetics, bioinformatics, and high-throughput sequencing technologies, improved the taxonomic identification of Argasidae family members, especially when considering specimens at different development stages, which remains a great difficulty for acarologists. These tools could provide important data and insights on the history and evolutionary relationships of argasids. To better understand these relationships, we sequenced and assembled the first complete mitochondrial genome of *Nothoaspis amazoniensis*. We used phylogenomics to identify the evolutionary history of this species of tick, comparing the data obtained with 26 complete mitochondrial sequences available in biological databases. The results demonstrated the absence of genetic rearrangements, high similarity and identity, and a close organizational link between the mitogenomes of *N*. *amazoniensis* and other argasids analyzed. In addition, the mitogenome had a monophyletic cladistic taxonomic arrangement, encompassed by representatives of the Afrotropical and Neotropical regions, with specific parasitism in bats, which may be indicative of an evolutionary process of cospeciation between vectors and the host.

## 1. Introduction

Argasid ticks have sexual dimorphism with a few differentiated stages, being morphologically classified into two subfamilies (Argasinae and Ornithodorinae), which are divided into six genera (*Antricola*, *Argas*, *Carios*, *Ornithodoros*, *Otobius*, and *Nothoaspis*) [1,2]. Due to poorly differentiated sexual dimorphism, tick species of Argasidae family are difficult to identify morphologically in adult stages and also in larval specimens. The difficulty of identification by morphological characteristics for each genus creates an important problem for the taxonomic classification of Argasidae species, which negatively impacts on faunistic and epidemiological studies [3,4]. According to Nava et al. [5], each life stage has a diagnostic morphological characteristic. The adults have a false shield or nothoaspis, a projecting hood covering the capitulum, a medial extension of palpal article I (flaps), a genital plate that extends from coxal I to IV, the absence of two setae on the internal margin of the flaps, a hypostome without denticles, a central pore in the base of hypostome, and a reticulate surface pattern on the posterior half of the nothoaspis in males. Specific to the nymph II stage, these characteristics are a small hood compared to the capitulum, short coxal setae, palpal flaps lacking setae on the internal margin, long hypostome, pointed with dentition 4/4 apically, and a cell-like configuration on the anterior half of the body. The nymph I stage is characterized by a hood, cell-like configuration covered on the dorsum side, a venter integument also with a cell-like configuration, and hypostome dentition 4/4 with apices that are ‘‘V’’-shaped. For the larval stage, the number and size of dorsal setae, and the shape of the scutum and hypostome, are the diagnostic characteristics. 

The notorious similarities between closely related species like *Ornithodoros setosus* are that the larvae from both species share a dorsal plate with similar size and shape, a basis capituli with lateral bulges, and the presence of reticulations in the capsule of Haller’s organ [5]. Also, nymph II from Brazil is morphologically similar to the nymph of *N. reddelli* described by Keirans et al. [6]. 

Conflicts between taxonomy and phylogenetic relationships in the Argasidae family have persisted for several years without a consensus about the species classification, often resulting in the same species being classified into more than one taxonomic group [4]. For Estrada-Peña et al. [3], the absence of morphological standards for identifying the members of this family provides a dubious genus classification for approximately 130 argasid species among the almost 200 argasid species that have been described worldwide. In addition, Burger et al. [7] point out that the taxonomic arrangement proposed thus far for argasids is limited because it was established before the use of molecular markers for the analysis of taxonomic relations. 

In recent years, high-throughput DNA sequencing technologies and bioinformatics tools have allowed the evolution of comparative and phylogenetic analysis of nuclear and mitochondrial genomes [8,9]. These tools have contributed in the identification of a large number of molecular markers and a better understanding of the phylogenetic relationships for many groups of organisms [8,10,11]. In this scenario, molecular phylogeny has been established as a valuable tool for genomic comparative analysis [12]. This approach, in a way, was consolidated due to the increasing number of genome sequences deposited in public databases [8,9,13]. 

Phylogenomic studies have provided better resolution for evolutionary reconstruction from genomic data when compared to phylogenetic approaches using a single gene or gene fragment sequences [14,15]. This approach allows for the comparative analysis of whole genomes, and, together with the increased sampling of new species, has contributed to the increased accuracy of phylogenetic methods to solve taxonomic conflicts [16,17,18,19]. It is worth noting that morphological taxonomy is a powerful tool for identification when correctly implemented. The combination of molecular and morphological analysis is indispensable for complete identification of a certain organism.

In this study, we report the first complete mitochondrial genome of *Nothoaspis amazoniensis* and perform a phylogenomic analysis of other species of Argasidae family to reconstruct their evolutionary history and provide data for future taxonomic studies on this family of ticks.

## 2. Materials and Methods

### 2.1. Mitochondrial Genome Sequences of Argasidae Species

A set of complete mitochondrial genome sequences of tick species from the Argasidae family was downloaded from the GenBank database (http://www.ncbi.nlm.nih.gov/Genbank) to perform the phylogenomic analysis (Table 1).

### 2.2. Sequencing of the Nothoaspis amazoniensis Mitochondrial Genome

#### 2.2.1. DNA Extraction and Quantification

An adult male *N. amazoniensis* tick, previously morphologically identified according to the taxonomy described by Nava et al. [5], was collected from a cave in Porto Velho Municipality, state of Rondônia, Brazil (08°37′59′′S, 63°57′29′′W), on 30 January 2010 and preserved in ethanol 70% for DNA extraction in 2015. The tick was decontaminated and the total DNA extracted using a QIAamp DNA Mini kit (Qiagen, Hilden, Germany) and quantified using a Quant-iT™ PicoGreen^®^ kit (Invitrogen, Carlsbad, CA, USA), according to the manufacturer’s recommendations.

#### 2.2.2. Library Preparation, Normalization, and Clustering

The genomic library (nuclear and mitochondrial DNA) of *N. amazoniensis* was prepared with the Nextera DNA Sample Prep kit (Illumina, San Diego, CA, USA), using approximately 50 ng of DNA. The quality assessment of the genomic library was performed using the Agilent 2100 Bioanalyzer system (Agilent Technologies, Santa Clara, CA, USA) and the DNA sample was prepared with initial concentration of 10 nM. An aliquot of this library containing 2 nM of DNA fragments was quantified through an absolute quantification curve using KAPA SYBR^®^ FAST qPCR (Kapa Biosystems, Wilmington, MA, USA) on a StepOne Real Time (Applied Biosystems, Foster City, CA, USA), according to the manufacturer’s recommendations.

The fragment indexing process was performed according to the manufacturer’s recommendations, using the HiSeq cBot Manifold (Illumina, San Diego, CA, USA) and the TrueSeq PE Cluster Kit v3—cBot HS reagents (Illumina, San Diego, CA, USA). Sixteen picomoles of the *N. amazoniensis* library were applied to the surface of the sequencing slide using the cBot Cluster Generation System (Illumina, San Diego, CA, USA) automated system.

#### 2.2.3. Library Sequencing

The library sequencing was performed on an Illumina HiSeq 2500 using the High Output mode. The sequencing run was carried out using paired-end and single-indexed adapters with a total of 209 cycles. The procedures were performed using the TrueSeq SBS Kit v3-HS-200 cycles (Illumina, San Diego, CA, USA), according to the manufacturer’s protocol. A total of 129,718,692 reads with sizes ranging from 85 to 101 nucleotides were produced.

### 2.3. Assembling and Functional Annotation of the N. amazoniensis Mitochondrial Genome

After checking the quality of sequence data using FastQC software version 0.11.1 [22], the reads were quality-trimmed (Q20 score) and length-filtered (75 nt) using Trimmomatic version 2.32 [23]. Fourteen percent of reads were filtered out by this processing, resulting in a dataset of 100,869,030 paired-end reads and 10,547,679 single reads with sizes ranging between 75 and 101 nucleotides.

The selected reads were used to assemble the mitochondrial genome sequence of the *N. amazoniensis* using the de novo assembly algorithm by CLC Genomics Workbench version 6.5.1 (Qiagen, Hilden, Germany). This assembly produced a contig containing 14,416 nt and 1,464,476 reads aligned with a coverage of 9727.28-fold.

Mitochondrial genes were predicted and annotated using Dual Organellar Genome Annotator (DOGMA) [24] with the default settings for mitochondrial invertebrate genes. Prediction and annotation of transfer RNA genes (tRNA) were also confirmed by ARWEN version 1.2 [25]. The predicted genes were compared to the reference genome of *N. amazoniensis* (KC_769595.1), the partial genome, and *Otobius megnini* (NC_023370). 

### 2.4. Comparative Genomics

The mitochondrial genome of *N. amazoniensis* was aligned to the mitochondrial sequences of other argasids using ClustalW [26]. A pairwise distance matrix was calculated from the alignment using MEGA 6.0 [27]. From this matrix the mean distance between the mitochondrial sequences of each genus of Argasidae family was calculated. A search for locally collinear blocks (LCBs) was performed using Mauve version 2.4.0 [28] to compare the mitochondrial genomes and identify possible structural variations, such as inversions, translocations, insertions, deletions, and duplications.

### 2.5. Phylogenetic Analysis

To perform the phylogenetic analysis, 27 complete sequences of mitochondrial genomes were selected: (i) the sequence of *N. amazoniensis*; (ii) 18 sequences of tick species of the Argasidae family (Table 1); (iii) five sequences of Metastriata (*Amblyomma cajennense*, *Bothriocroton concolor*, *Haemaphysalis flava*, *Rhipicephalus sanguineus* and *Dermacentor nitens*) and one sequence of Prostriata (*Ixodes uriae*), as representatives of the Ixodidae family; (iv) one sequence of the *Nuttalliella namaqua*, as a monotypic representative of the Nuttalliellidae family; and (v) one sequence of the *Varroa destructor*, selected as an outgroup to root the phylogenetic tree.

The phylogenetic tree was inferred by the Bayesian Inference (BI) method using MrBayes version 3.11 [29,30]. To expedite the construction of the phylogenetic tree, the GTR + I + G was selected as the best-fit nucleotide substitution model using jModelTest version 2.1.10 [31]. The BI tree was calculated using the Bayesian Markov Chain Monte Carlo (MCMC) in four independent runs with one million generations and a sampling frequency of 1000. We set the burn-in to 10% to generate the consensus phylogenetic tree. 

## 3. Results

### 3.1. Characterization of the Nothoaspis amazoniensis Mitogenome

**Figure 1:**
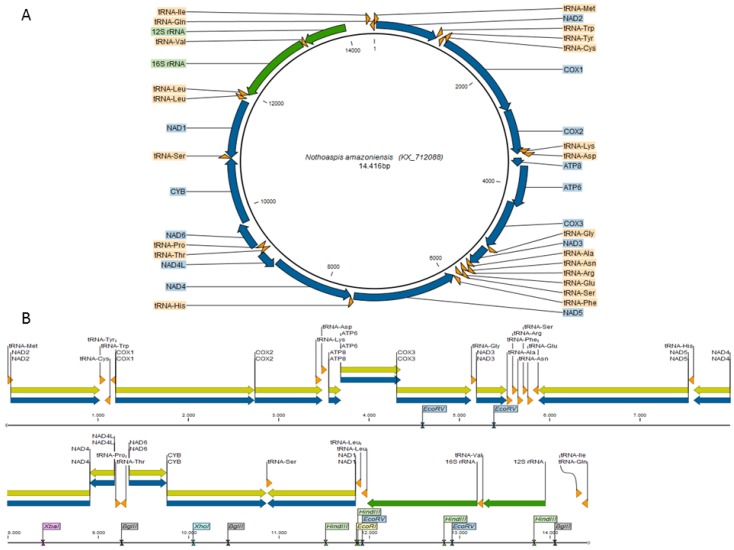
Mitochondrial genome map of *Nothoaspis amazoniensis*. (**A**) Circular representation; (**B**) linear representation. Thirty-seven genes (blue) and their respective CDS (yellow), two rRNA genes (green), and 22 tRNA genes (orange) are highlighted. The restriction enzyme sites are highlighted in the linear representation. The control region is located between the 12S rRNA and tRNA-IIe genes.

The complete mitochondrial genome of *N. amazoniensis* is available in GenBank under the accession number KX712088. The circular sequence is 14,416 nt in length (Figure 1A) and contains 37 genes, 13 coding DNA sequences (CDS), two ribosomal genes (12S rRNA and 16S rRNA), and 22 transfer RNA (tRNA) genes. Twenty-two genes are located in the heavy strand (H) and 15 genes in the light strand (L) (Table S1).

Thirteen restriction sites were found in the *N. amazoniensis* mitogenome (Figure 1B and Table S2). Overlapping regions were found between pairs of 22 genes, corresponding to a total of 265 nt. The longest overlap (66 nt) was found between the COX2 and tRNA-Lys genes (Tables S1 and S3). In addition to the non-coding control region (341 nt) typical of argasid ticks [7,20,32], other non-coding intergenic regions were also identified in the *N. amazoniensis* mitogenome (Tables S1 and S4).

The nucleotide sequence of the *N. amazoniensis* mitogenome contains 39.34% adenine, 33.59% thymine, 8.13% guanine, and 18.94% cytosine. The AT content is higher (72.93%) than the GC content (27.07%), which is a typical feature of the mitochondrial genome of metazoan arthropods [33] and similar to the GC data obtained in the phylogenetic studies of Burger et al. [7] for ticks of the Argasidae family. 

### 3.2. Comparative Genomics

The pairwise distance matrix showed high conservation among the analyzed mitogenomes, confirming that the species of the Argasidae family are genetically close, with an overall identity of 69.64% (Table S5). Argasinae and Ornithodorinae species showed an average identity of 77.33% and 73.65%, respectively. Only a single locally collinear block (LCB) was identified among the analyzed mitogenomes, confirming that Argasidae genomes are structurally conserved without genomic rearrangements (Figure S1).

### 3.3. Phylogenetic Analysis

**Figure 2:**
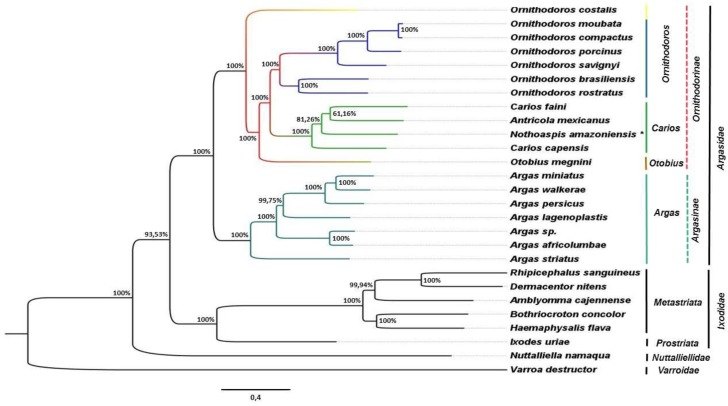
Evolutionary relationships among ticks of the Argasidae, Ixodidae, and Nuttalliellidae families. The major-rule consensus phylogenetic tree was generated by Bayesian Inference analysis of 27 complete sequences of mitochondrial genomes. The posterior probability values (PP) (expressed as percentages), calculated using the best trees found by MrBayes, are shown beside each node. * Species of *N. amazoniensis* analyzed in this study.

In the phylogenetic tree (Figure 2), the mitochondrial genomes of species of the Argasidae, Ixodidae (including Metastriata and Prostriata), and Nuttalliellidae families were clustered in distinct monophyletic clades supported by high values of posterior probabilities (PP). Also, the topology of the phylogenetic tree suggests a common ancestry for Argasidae and Ixodidae.

Within the Argasidae clade, Argasinae and Ornithodorinae subfamilies were also clustered into distinct monophyletic clades (PP = 100%). Only mitochondrial genomes of species of the *Argas* genus were clustered in the Argasinae clade. On the other hand, the mitogenomes of species of the genus *Antricola*, *Carios*, *Nothoaspis*, *Ornithodoros*, and *Otobius* were clustered together in the Ornithodorinae clade.

In the Ornithodorinae clade, we proposed three monophyletic groups (PP = 100%) for the genera *Carios*, *Ornithodoros*, and *Otobius*, with the species *Ornithodoros costalis* remaining unassigned. We also proposed a monophyletic clade for the genus *Carios* (PP = 100%), including the species *Carios capensis* and *C. faini*, together with *Antricola mexicanus* and *N. amazoniensis*.

## 4. Discussion

The mitochondrial genome of *N. amazoniensis* has characteristics of metazoans and arthropods [34] and shares the same features found in the mitogenomes of ticks of the Argasidae family [7,20,21]. In addition, the analysis of collinearity and synteny showed a high structural conservation between the mitogenome of *N. amazoniensis* and other genomes of argasid ticks. This structural conservation among the mitochondrial genomes of argasids would be an advantage for population studies, as proposed by Nava et al. [4]. In addition, Shao et al. [20] emphasized that the synteny of mitochondrial genes of argasid ticks originated from ancestral arthropods and remained unchanged for more than 400 million years.

The lack of a consensus in the interpretations of the taxonomic and evolutionary process of the Argasidae family has resulted in different taxonomic proposals for tick species of this family [4]. Among these proposals, we highlight four taxonomic arrangements: Soviet [35,36], American [2,37], French [38,39], and cladistic [40]. The resulting phylogenetic tree herein agrees to the cladistic hypothesis proposed by Klompen and Oliver [40].

Klompen and Oliver [40] used morphological differentiation by way of 83 characteristics with the development and behavior observations of specimens of the Argasidae family to establish their cladistic hypothesis, emphasizing that the recognition of monophyletic taxa would be one of the strong points of this theory. For Rieppel [41], the monophyletic taxa structure (natural group) is extremely important for phylogenetic systematics, which is the process ensuring the success of evolutionary identification methods, whose function is to verify and establish the natural affinity between the analyzed organisms and constitute the hierarchy of grouping.

In the phylogenetic tree, all mitochondrial genomes of species of the Argasinae subfamily were included in a monophyletic clade supported by high values of posterior probabilities (PP); this condition is consistent with the monophyly for the *Argas* genus proposed by Klompen and Oliver [40]. The monophyletic group of Argasinae was characterized by the natural grouping of subgenus *Persicargas* (*Argas miniatus*, *A. persicus*, and *A. walkerae*) [40]. However, it was not possible to confirm the monophyletic groups of these subgenera in the phylogenetic tree and we propose the taxonomic classification of the species as being only part of the *Argas* genus, which could be explained by the unknown condition of *Argas* sp. Springbok-QMS95171 in relation to the absence of a more precise taxonomic definition for a subgenus.

In the Ornithodorinae subfamily, the *Ornithodoros* genus was characterized as a monophyletic group that includes the subgenera *Pavlovskyella* and *Ornithodoros* [40]. This condition was also observed in the phylogenetic tree, which showed monophyletic clades for the *Ornithodoros* genus (PP = 100%) and for the subgenera *Pavlovskyella* (*Ornithodoros brasiliensis* and *O. rostratus*; PP = 100%) and *Ornithodoros* (*O. compactus*, *O. moubata*, *O. porcinus*, and *O. savignyi*; PP = 100%). However, *O. costalis* was placed outside of the monophyletic clade of the *Ornithodoros* genus in the phylogenetic tree and its taxonomic classification in the Ornithodorinae subfamily should be reviewed.

*Otobius megnini* was the only representative specie of the *Otobius* genus in the phylogenetic tree and was placed as a sister group of the *Carios* and *Ornithodoros* genera in the Ornithodorinae subfamily, as previously proposed by Burger et al. [7], Clifford et al. [37], and Klompen and Oliver [40].

The adoption of the Carios genus would still be controversial among taxonomists of ticks [42]. According to Camicas et al. [39], Estrada-Peña et al. [43], and Venzal et al. [44], it is extremely important to incorporate a greater amount of information related to life history, morphological and molecular characteristics, and their interaction for the establishment of the *Carios* genus. From the phylogenetic tree, we propose a monophyletic clade (PP = 100%) for the *Carios* genus that groups the species *Carios capensis* and *C. fani* together with *Antricola mexicanus* and *N. amazoniensis*. We also observed a biogeographical component in this clustering, with *C. capensis* (Afrotropical region) separated from the species *A. mexicanus*, *C. fani* and *N. amazoniensis* (Neotropical region) [5,45,46]. The joining of the subgenera *Carios*, *Chiropterargas*, *Alectorobius*, *Reticulinasus*, *Subparmatus*, *Parantricola*, *Antricola*, and *Nothoaspis* in the same *Carios* genus was also previously proposed by Klompen and Oliver [40].

The phylogenomic analysis performed herein contributes to a better understanding of the evolutionary relationships among the species of the Argasidae family, confirming the structural conservation of mitochondrial genomes and corroborating the taxonomic classification recommended by Klompen and Oliver [40]. However, it is necessary to increase the availability of Argasidae mitochondrial genomes and data about life stages, morphology, and molecular characteristics in order to highlight the conflicts between taxonomy and phylogenetic relationships. According to Estrada-Peña et al. [3] and Nava et al. [4], the use of the molecular taxonomy associated with traditional morphological cataloging, the so-called “Classical Taxonomy,” is useful to obtain more homogeneous criteria in the establishment of a more precise taxonomic arrangement for the Argasidae family.

## 5. Conclusions

The complete mitochondrial genome sequence of *N. amazoniensis* presented here adds new information to the repertoire of argasid mitogenomes. According to the phylogenomic analysis, *N. amazonensis* is a member of the *Carios* genus in the Ornithodorinae subfamily of the Argasidae family. This genus included ticks of the biogeographic Afrotropical and Neotropical regions, with specific parasitism in bats, emphasizing the characterization of a potentially wide coevolution state between parasite and its host, a fact of great importance for the evolution of the species in these varied ecosystems.

## Figures and Tables

**Table 1:** Mitochondrial genomes of argasids selected for this study.

Species	ID GenBank (genome mt)	Reference
*Antricola mexicanus*	NC_023340.1	Burger et al., 2014 [7]
*Argas africolumbae*	NC_019642.1	-
*Argas lagenoplastis*	NC_023369.1	Burger et al., 2014 [7]
*Argas miniatus*	NC_023371.1	Burger et al., 2014 [7]
*Argas persicus*	NC_029174.1	-
*Argas* sp. SpringbokSA-QMS95171	KC_769588.1	Burger et al., 2014 [7]
*Argas striatus*	NC_029175.1	-
*Argas walkerae*	NC_029176.1	-
*Carios capensis*	NC_005291.1	-
*Carios faini*	NC_029177.1	-
*Ornithodoros brasiliensis*	NC_023373.1	Burger et al., 2014 [7]
*Ornithodoros compactus*	NC_029178.1	-
*Ornithodoros costalis*	NC_029179.1	-
*Ornithodoros moubata*	NC_004357.1	Shao et al., 2004 [20]
*Ornithodoros porcinus*	NC_005820.1	Mitani et al.,2004 [21]
*Ornithodoros* *rostratus*	NC_023372.1	Burger et al., 2014 [7]
*Ornithodoros savignyi*	NC_029180.1	-
*Otobius megnini*	NC_023370.1	Burger et al., 2014 [7]

## Supplementary Materials

The following are available online at http://www.mdpi.com/2306-7381/5/2/37/s1, Figure S1: Distance matrices and identity of the complete mitogenome of tick belonging to Argasidae family, Table S1: Gene order, gene position, intergenic regions, gene overlap and DNA transcription direction strand in the mitochondrial genome of *Nothoaspis amazoniensis*, Table S2: Cleavage sites for restriction enzymes in the mitogenome of *Nothoaspis amazoniensis*, Table S3: Gene overlaps for the mitogenome of *Nothoaspis amazoniensis*, Table S4: Non-coding intergenic regions in the mitogenome of *Nothoaspis amazoniensis*, Table S5: Distance matrices and identity of the complete mitogenome of tick belonging to Argasidae family.
