# Supplementary material for: The Nothoaspis amazoniensis Complete Mitogenome: A Comparative and Phylogenetic Analysis
Source: Vet Sci. 2018 Mar 27;5(2):37. doi: 10.3390/vetsci5020037 (PMC6024882; doi:10.3390/vetsci5020037)
Supplement: Supplementary file 1 [file vetsci-05-00037-s001.pdf]

Table S1. Gene order, gene position, intergenic regions, gene overlap and DNA transcription direction strand in the mitochondrial genome of *Nothoaspis amazoniensis.*

Genes	Gene position and intergenic regions*		Gene size and intergenic regions (pb)	Strand direction
//* tRNA-Met	1	61[b]	61	Positive
NAD2	32[b]	1021	990	Positive
tRNA-Trp	1020	1082[b]	63	Positive
tRNA-Cys	1075[b]	1135	61	Negative
tRNA-Cys / tRNA-Tyr[a]	1136[a]	1137[a]	2	-
tRNA-Tyr	1138	1199[b]	62	Negative
COX1	1192[b]	2730	1539	Positive
COX1 / COX2[a]	2731[a]	2733[a]	3	-
COX2	2734	3483[b]	750	Positive
tRNA-Lys	3410[b]	3475[b]	66	Positive
tRNA-Asp	3474[b]	3533	60	Positive
tRNA-Asp / ATP8[a]	3534[a]	3551[a]	18	-
ATP8	3552	3689[b]	138	Positive
ATP6	3683[b]	4351[b]	669	Positive
COX3	4303[b]	5130[b]	828	Positive
tRNA-Gly	5130[b]	5189[b]	60	Positive
NAD3	5187[b]	5525	339	Positive
tRNA-Ala	5526	5587[b]	62	Positive
tRNA-Arg	5587[b]	5644	58	Positive
tRNA-Asn	5645	5706[b]	62	Positive
tRNA-Ser	5704[b]	5754	51	Positive
tRNA-Glu	5755	5815[b]	61	Positive
tRNA-Phe	5814[b]	5874	61	Negative
NAD5	5875	7537[b]	1663	Negative
tRNA-His	7535[b]	7594[b]	60	Negative
NAD4	7593[b]	8909[b]	1317	Negative
NAD4L	8903[b]	9181	279	Negative
NAD4L / tRNA-Thr[a]	9182[a]	9188[a]	7	-
tRNA-Thr	9189	9247	59	Positive
tRNA-Pro	9248	9308	61	Negative
tRNA-Pro / NAD6[a]	9309[a]	9336[a]	28	-
NAD6	9337	9756[b]	420	Positive
CYB	9756[b]	10859	1104	Positive
CYB / tRNA-Ser[a]	10860[a]	10861[a]	2	-

tRNA-Ser	10862	10924[b]	63	Positive
NAD1	10872[b]	11849[b]	978	Negative
tRNA-Leu	11847[b]	11906	60	Negative
tRNA-Leu / tRNA-Leu[a]	11907[a]	11910[a]	4	-
tRNA-Leu	11911	11975[b]	65	Negative
16S rRNA	11975[b]	13195	1221	Negative
tRNA-Val	13196	13258	63	Negative
12S rRNA	13259	13949	691	Negative
12S rRNA / tRNA-Ile[a] (CR)	13950[a]	14290[a]	341	-
tRNA-Ile	14291	14353[b]	63	Positive
tRNA-Gln //*	14351[b]	14416	66	Negative

[a] Intergenic regions; [b] Gene overlapping; Control Region (CR); //*: Circular link.

Table S2. Cleavage sites for restriction enzymes in the mitogenome of Nothoaspis amazoniensis.

Cleavage sites	Position of the cleavage between bases	Type of cleavage	Gene/Intergenic Interval
EcoRV	4592-4593	B	COX3
EcoRV	5381- 5382	B	NAD3
Xbal	8385-8386 / 8389-8390	A	NAD4
BgIII	9255-9256 / 9259-9260	A	tRNA-Pro
Xhol	10048-10049 / 10052-10053	A	CYB
BgIII	10433-10434 / 10437-10438	A	CYB
HindIII	11515-11516 / 11519-11520	A	NAD1
EcoRI	11855-11856 / 11859-11860	A	tRNA-Leu
HindIII	11871-11872 / 11875-11876	A	tRNA-Leu
EcoRV	11921-11922	B	tRNA-Leu
HindIII	12825-12826 / 12829-12830	A	16S rRNA
EcoRV	12915-12916	B	16S rRNA
HindIII	13821-13822 / 13825-13826	A	12S rRNA
BgIII	14051-14052 / 14055-14056	A	12S rRNA / tRNA-Ile (CR)

A: symmetrically located around the axis of symmetry; B: in the axis of symmetry; CR: Control Region.

Table S3. Gene overlaps for the mitogenome of Nothoaspis amazoniensis.

Genes	Overlapping passages	bp
tRNA-Met / NAD2	32-61	30
NAD2 / tRNA-Trp	1020-1021	2
tRNA-Trp / tRNA-Cys	1075-1082	8
tRNA-Tyr / COX1	1192-1199	8
COX2 / tRNA-Lys	3410-3475	66
tRNA-Lys / tRNA-Asp	3474-3475	2

COX2 / tRNA-Asp	3474-3483	10
ATP8 / ATP6	3683-3689	7
ATP6 / COX3	4303-4351	49
COX3 / tRNA-Gly	5130	1
tRNA-Gly/ND3	5187-5189	3
tRNA-Ala / tRNA-Arg	5587	1
tRNA-Asn / tRNA-Ser	5704-5706	3
tRNA-Glu / tRNA-Phe	5814-5815	2
NAD5 / tRNA-His	7535-7537	3
tRNA-His / NAD4	7593-7594	2
NAD4 / NAD4L	8903-8909	7
NAD6 / CYB	9756	1
tRNA-Ser / NAD1	10872-10924	53
NAD1 / tRNA-Leu	11847-11849	3
tRNA-Leu / 16S rRNA	11975	1
tRNA-Ile / tRNA-Gln	14351-14353	3

Table S4. Non-coding intergenic regions in the mitogenome of Nothoaspis amazoniensis.

**Genes**	**Intergenic regions intervals**	**bp**
tRNA-Cys / tRNA-Tyr	1136-1137	2
COX1 / COX2	2731-2733	3
tRNA-Asp / ATP8	3534-3551	18
NAD4L / tRNA-Thr	9182-9188	7
tRNA-Pro / ND6	9309-9336	28
CYB / tRNA-Ser	10860-10861	2
tRNA-Leu / tRNA-Leu	11907-11910	4
12S rRNA / tRNA-Ile [(CR)]	13950-14290	341

CR: Control Region.

Table S5. Distance matrices and identity of the complete mitogenome of tick belonging to Argasidae family.

			p-distance matrix	1	2	3	4	5	6	7	8	9	10	11	12	13	14	15	16	17	18
Family *Argasidae*	Subfamily Ornithodorinae	1	*Nothoaspis amazoniensis*																		
		2	*Antricola mexicanus*	0,229																	
		3	*Carios faini*	0,231	0,219																
		4	*Carios capensis*	0,240	0,233	0,227															
		5	*Ornithodoros brasiliensis*	0,266	0,262	0,265	0,261														
		6	*Ornithodoros rostratus*	0,266	0,263	0,261	0,257	0,225													
		7	*Otobius megnini*	0,276	0,267	0,270	0,278	0,269	0,261												
		8	*Ornithodoros savignyi*	0,288	0,291	0,295	0,296	0,268	0,274	0,293											
		9	*Ornithodoros moubata*	0,289	0,284	0,281	0,282	0,269	0,270	0,278	0,223										
		10	*Ornithodoros porcinus*	0,290	0,283	0,283	0,281	0,266	0,269	0,282	0,227	0,172									
		11	*Ornithodoros costalis*	0,290	0,288	0,280	0,284	0,271	0,275	0,279	0,299	0,288	0,293								
		12	*Ornithodoros compactus*	0,291	0,288	0,282	0,285	0,270	0,271	0,279	0,223	0,035	0,171	0,287							
	Subfamily Argasinae	13	*Argas striatus*	0,294	0,291	0,289	0,295	0,295	0,287	0,290	0,321	0,297	0,302	0,298	0,300						
		14	*Argas walkerae*	0,302	0,300	0,296	0,300	0,294	0,292	0,292	0,316	0,306	0,308	0,298	0,306	0,251					
		15	*Argas* sp. SpringbokSA -QMS95171	0,303	0,301	0,296	0,306	0,305	0,297	0,293	0,320	0,314	0,310	0,304	0,314	0,256	0,236				
		16	*Argas miniatus*	0,305	0,301	0,294	0,302	0,298	0,291	0,296	0,322	0,310	0,307	0,302	0,310	0,253	0,160	0,235			
		17	*Argas lagenoplastis*	0,308	0,306	0,299	0,309	0,303	0,296	0,299	0,319	0,309	0,316	0,304	0,308	0,255	0,229	0,232	0,231		
		1	*Argas*	0,309	0,304	0,303	0,307	0,304	0,299	0,295	0,319	0,313	0,312	0,304	0,313	0,255	0,234	0,133	0,238	0,233	

	18	*africolumbae*																	
	19	*Argas persicus*	0,309	0,301	0,298	0,306	0,304	0,296	0,301	0,320	0,310	0,315	0,304	0,309	0,260	0,174	0,241	0,180	0,236	0,238

		Identity matrix	1	2	3	4	5	6	7	8	9	10	11	12	13	14	15	16	17	18
Subfamily *Ornithodorinae*	1	*Nothoaspis amazoniensis*																		
	2	*Antricola mexicanus*	77,08 %																	
	3	*Carios faini*	76,87 %	78,14 %																
	4	*Carios capensis*	75,97 %	76,67 %	77,32 %															
	5	*Ornithodoros brasiliensis*	73,38 %	73,80 %	73,52 %	73,93 %														
	6	*Ornithodoros rostratus*	73,39 %	73,69 %	73,90 %	74,30 %	77,53 %													
	7	*Otobius megnini*	72,43 %	73,30 %	72,95 %	72,20 %	73,08 %	73,86 %												
	8	*Ornithodoros savignyi*	71,15 %	70,93 %	70,45 %	70,44 %	73,19 %	72,62 %	70,69 %											
	9	*Ornithodoros moubata*	71,09 %	71,56 %	71,90 %	71,80 %	73,05 %	72,96 %	72,24 %	77,71 %										
	10	*Ornithodoros porcinus*	71,03 %	71,67 %	71,71 %	71,94 %	73,36 %	73,12 %	71,80 %	77,29 %	82,84 %									
	11	*Ornithodoros costalis*	71,03 %	71,17 %	72,04 %	71,64 %	72,88 %	72,47 %	72,09 %	70,12 %	71,19 %	70,69 %								
	12	*Ornithodoros compactus*	70,93 %	71,16 %	71,83 %	71,52 %	73,01 %	72,89 %	72,12 %	77,73 %	96,54 %	82,89 %	71,27 %							
Family *Argasidae*, Subfamily Argasinae	13	*Argas striatus*	70,62 %	70,93 %	71,11 %	70,45 %	70,52 %	71,31 %	71,05 %	67,91 %	70,29 %	69,79 %	70,20 %	70,05 %						
	14	*Argas walkerae*	69,82 %	70,03 %	70,36 %	70,04 %	70,57 %	70,78 %	70,75 %	68,38 %	69,41 %	69,20 %	70,16 %	69,41 %	74,86 %					
	15	*Argas* sp. SpringbokSA -QMS95171	69,71 %	69,94 %	70,36 %	69,40 %	69,52 %	70,33 %	70,69 %	67,98 %	68,58 %	68,97 %	69,57 %	68,60 %	74,35 %	76,43 %				
	16	*Argas miniatus*	69,47 %	69,86 %	70,56 %	69,79 %	70,23 %	70,90 %	70,35 %	67,77 %	68,99 %	69,28 %	69,78 %	68,99 %	74,69 %	84,04 %	76,51 %			

17	*Argas lagenoplastis*	69,19 %	69,36 %	70,10 %	69,09 %	69,68 %	70,39 %	70,13 %	68,14 %	69,08 %	68,41 %	69,60 %	69,16 %	74,51 %	77,12 %	76,84 %	76,90 %		
18	*Argas africolumbae*	69,14 %	69,61 %	69,71 %	69,28 %	69,63 %	70,08 %	70,53 %	68,13 %	68,74 %	68,79 %	69,58 %	68,72 %	74,48 %	76,55 %	86,72 %	76,17 %	76,72 %	
19	*Argas persicus*	69,05 %	69,89 %	70,25 %	69,36 %	69,56 %	70,36 %	69,88 %	67,98 %	68,96 %	68,51 %	69,57 %	69,09 %	74,01 %	82,57 %	75,94 %	81,97 %	76,39 %	76,22 %

Group	Average distance and standard deviation	Average identity
	0,279 ± 0,002	72,10%

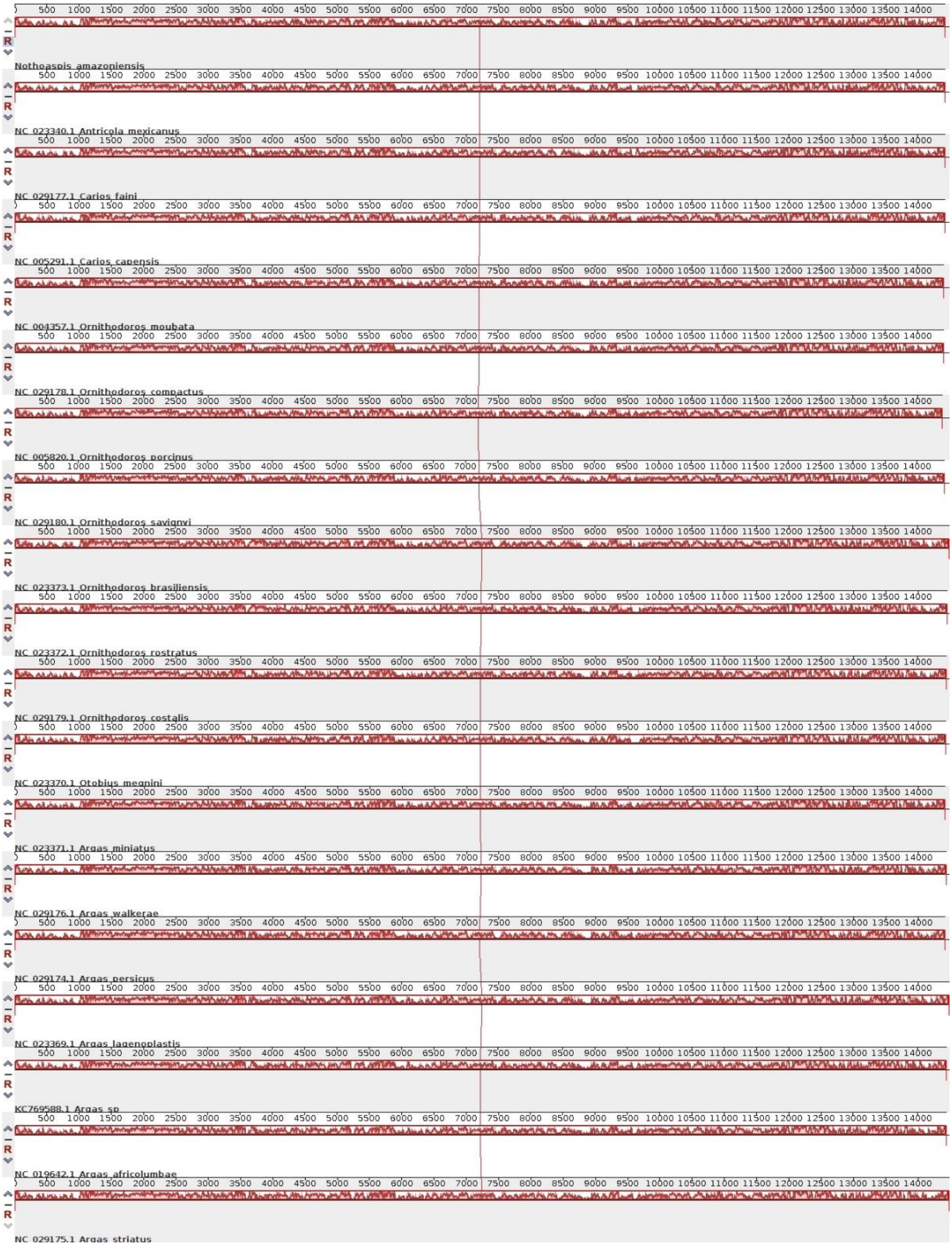

Figure S1. Comparative and organizational alignment of complete mitochondrial genomes of the Argasidae family. Red connects conserved blocks.
